# Development of avalanches and efficient communication in neuronal networks

**DOI:** 10.1186/1471-2202-15-S1-P31

**Published:** 2014-07-21

**Authors:** Jean-Philippe Thivierge, Joseph S Tauskela

**Affiliations:** 1 School of Psychology and Center for Neural Dynamics, University of Ottawa, Ottawa, Ontario K1N 6N5, Canada; 2 Human Health Therapeutics, National Research Council, Ottawa, Ontario K1A 0R6, Canada

## 

**Figure 1:**
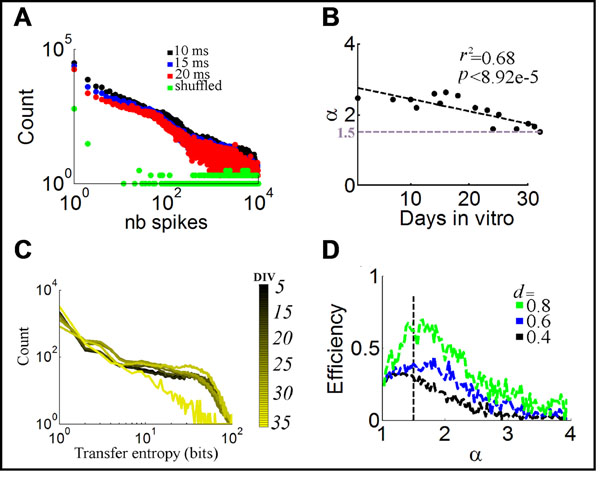
Avalanches and information transfer over the course of neuronal development. **A.** Distribution of spikes per avalanche (28 days in vitro), with time-bins of different durations. **B.** Gradual decrease in the slope of best-fitting power law during development. **C.** Alteration in the distribution of transfer entropy over development. **D.** Network efficiency in artificial data derived from power laws with different scaling exponents (α) and densities (*d*).

Over the course of neural development, changes in the morphology of the neural tissue are accompanied by changes in patterns of activity. One form of activity that is highly studied in cultured cortical networks is neuronal avalanches, characterized by bursts whose distribution follows a power law. Despite a detailed characterization of neuronal avalanches, much remains unknown about their gradual emergence during development [1]. Here, we examined 643,039 avalanches in 15 cortical cultures grown to 35 days in vitro on microelectrode arrays. We employed maximum likelihood estimation to evaluate the fit of a power law to the duration and amplitude of avalanches at different points during development (Figure 1A). The slope of the best-fitting power law followed a gradual trend from α≈2.3 in early recordings to α≈1.5 around 25 days in vitro (Figure 1B).

To examine the implications of this trend, we evaluated communication between pairs of neurons using a measure of transfer entropy that quantifies the amount of information (in bits) in a neuron found in the past history of another neuron [2]. Transfer entropy increased over development, and its distribution followed a power law with a slope approaching α≈1.5 towards 30 days in vitro (Figure 1C). Next, we generated artificial data whose distribution matched the power law of transfer entropy observed experimentally. Using graph-theoretical analyses, we show that a power law with an exponent of α≈1.5 maximizes network efficiency by facilitating rapid communication across neurons while minimizing the overall traffic burden (Figure 1D).

In sum, this study links the gradual development of power law scaling with increased communication efficiency in networks of cortical neurons. Incremental changes in network dynamics suggest that power scaling of avalanches and communication are shaped concurrently over the course of in vitro development, and may arise from a common origin. This developmental trend poses a particular challenge for computational models of avalanches that typically focus on the endpoint of development [3], and therefore merits the attention of further experimental and theoretical work.
